# Design of FitFor2 study: the effects of an exercise program on insulin sensitivity and plasma glucose levels in pregnant women at high risk for gestational diabetes

**DOI:** 10.1186/1471-2393-9-1

**Published:** 2009-01-05

**Authors:** Nicolette Oostdam, Mireille NM van Poppel, Elisabeth MW Eekhoff, Maurice GAJ Wouters, Willem van Mechelen

**Affiliations:** 1 Department of Public and Occupational Health, EMGO-Institute, VU University Medical Centre, Amsterdam, the Netherlands; 2 Body@Work Research Centre Physical Activity, Work and Health, TNO-VU, Amsterdam and Hoofddorp, the Netherlands; 3 Department of Endocrinology, VU University Medical Centre, Amsterdam, the Netherlands; 4 Department of Obstetrics and Gynaecology, VU University Medical Centre Amsterdam, the Netherlands

## Abstract

**Background:**

Pregnancy is a period in the life of women that is often associated with decreased daily physical activity and/or exercise. However, maintaining adequate levels of daily physical activity during pregnancy is important for mother and child. Studies suggest that moderate daily physical activity and exercise during pregnancy are associated with reductions in the risk of gestational diabetes mellitus (GDM). However, at present, physical activity is not routinely advised to pregnant women at risk for gestational diabetes in the Netherlands. In FitFor2-study we aim to assess whether an exercise program can improve insulin sensitivity and fasting plasma glucose levels of women at high risk for gestational diabetes, assuming that this will lower their risk of gestational diabetes.

**Methods:**

The FitFor2-study is a randomised controlled trial. Women who visit one of the participating hospitals or midwifery practices and who are at risk for gestational diabetes are eligible to participate. After baseline measurement they are randomly allocated to in the intervention or control group. The intervention group receives an exercise program twice a week in addition to usual care. The exercise program consist of aerobic and strength exercises and takes place under close supervision of a physiotherapist. Data are collected at 15, 24 and 32 weeks of pregnancy and 12 weeks after delivery. Primary maternal outcome measures are fasting plasma glucose and relative increase in insulin resistance. Primary neonatal outcome is birth weight. Secondary outcome measures are: maternal serum triglycerides, HDL, cholesterol, HbA1c, maternal weight gain during pregnancy, maternal physical activity level, foetal growth.

**Discussion:**

If the FitFor2 intervention program proves to be effective, obstetricians and midwives should refer women at risk for GDM to a special exercise program. Exercise programs for pregnant women under supervision of an experienced trainer are already available in the Netherlands, and these programs could be adjusted easily for this target group. Furthermore, the costs of these programs should be refunded by including them in the basic health care cost reimbursement schemes.

**Trial registration:**

NTR1139

## Background

In 2007, the Centers for Disease Control and Prevention (CDC) and the American College of Sports Medicine (ACSM) have recommended that all healthy adults aged 18 to 65 yr accumulate moderate-intensity aerobic (endurance) physical activity for a minimum of 30 minutes on five days each week or vigorous-intensity aerobic physical activity for a minimum of 20 minutes on three days each week. Combinations of moderate- and vigorous-intensity activity can be performed to meet this recommendation [1]. Physical inactivity is a major risk factor for cardiovascular and obesity-related diseases, such as hypertension, atherosclerosis, and diabetes mellitus type 2 [2].

Pregnancy is a period in the life of women that is often associated with decreased daily physical activity and decreased participation in sports and exercise [3]. Ning et al. reported that 23% of previously active women ceased to engage in exercise completely during pregnancy [4]. Factors that might influence the level of physical activity during pregnancy are, for instance, early pregnancy symptoms, such as nausea and fatigue or the perception that physical activity during pregnancy is risky to maternal or foetal health [3]. In the absence of either medical or obstetric complications, the American College of Obstetricians and Gynaecologists (ACOG) recommended 30 minutes or more of moderate exercise on most, if not all, days of the week for pregnant women.

A reduction in daily physical activity levels and lack of exercise is not the only change during pregnancy. Many physiological changes occur: in body weight, the vascular system, and in hormonal and energy balance. Shifts in maternal metabolism result in an increased insulin resistance during pregnancy. In women with a suboptimal beta-cell function, the increase in insulin secretion may not be sufficient to compensate the increased insulin resistance, resulting in gestational diabetes mellitus (GDM) [5]. GDM is defined as carbohydrate intolerance of varying degrees of severity with onset or first recognition during pregnancy and disappearance after pregnancy. GDM is associated with an increased maternal risk for other pregnancy-related complications, such as pre-eclampsia, postpartum haemorrhage, and with an increased risk for developing type 2 diabetes after pregnancy [6]. It also puts the infant at risk, since gestational diabetes is associated with an increased risk for macrosomia, jaundice and birth trauma [6]. Later in life, children of gestational diabetic mothers have an increased risk for obesity, abnormal glucose tolerance, and type 2 diabetes [6].

In Western countries, the most common treatment for women with GDM is dietary advice and secondly insulin treatment. Although regular daily physical activity and/or exercise is an established therapeutic adjunct in type 2 diabetes mellitus in non-pregnant adults, it is not routinely offered to women with GDM. This is because physicians and the mothers worry about several maternal physical responses during exercises that may be negatively affect the foetus [8]. Potential adverse effects could be growth retardation, premature labour, foetal trauma [7]. However, no such ill-effects of moderate physical activity or exercise have been found in the literature [7-10].

The importance of regular physical activity for glycaemic control in women with GDM has already been shown repeatedly. The increased insulin resistance normally occurring during pregnancy can be reduced by increased levels of moderate intensity daily physical activity [11,12]. Already in 1989, Jovanovic-Peterson et al. [13] showed in a clinical trial that glycaemic control in women with GDM was improved after treatment with an exercise program, similar to improvements obtained with pharmacological therapies. These results were confirmed later by others [14-16]. However, most of these trials studied the effects of a short term exercise program (i.e. a single bout or only several weeks). Studies on longer lasting exercise programs, especially those continuing into the third trimester of pregnancy, are currently lacking. Furthermore, most studies concerned the treatment, and not the prevention of GDM.

Only case-control and cohort studies are available as evidence for a positive effect of regular physical activity and/or exercise in the prevention of GDM [17-19]. However, there are no reasons to suggest that the underlying mechanisms for GDM are any different than for type 2 diabetes mellitus in non-pregnant individuals. Since daily physical activity and/or exercise has been shown to be effective in the prevention of type 2 diabetes in high-risk adults [20], it is plausible that it will also be an effective strategy for the prevention of GDM.

So, based on the literature, increasing levels of daily physical activity and/or participation in an exercise program during pregnancy may be an effective strategy for the prevention of GDM. However, at the moment, such a regime is not routinely advised or prescribed for women at risk for GDM in the Netherlands.

Consequently, in the FitFor2-study the effects of an exercise program for women with an increased risk for GDM on fasting blood glucose levels, insulin resistance, and foetal birth weight in women with an increased risk for GDM will be determined. The overall aim of the FitFor2 study is to assess whether an exercise program can improve insulin sensitivity and fasting plasma glucose levels of women at high risk for gestational diabetes, assuming that this will lower their risk of GDM. Secondary objectives are a cost-effectiveness analysis and a process evaluation (i.e. assessing the compliance with the exercise program and what factors contribute to the success or failure of the program). The aim of this paper is to describe the design of a randomised controlled trial, the FitFor2 study.

## Methods

### Study design

**Figure 1:**
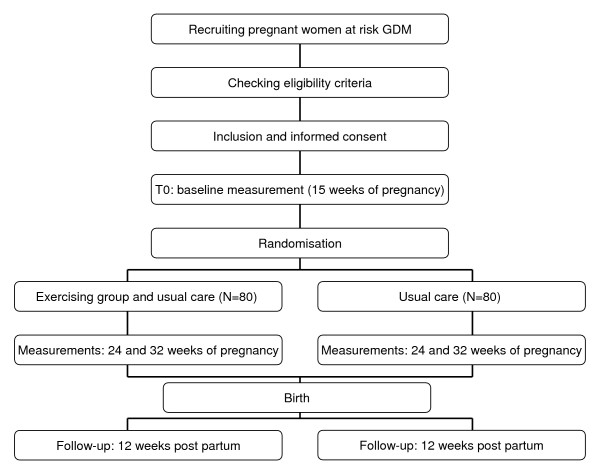
**Study design**.

This study is designed as a randomised controlled trial (RCT), to assess whether an exercise program improves insulin sensitivity and fasting plasma glucose levels of women at high risk for gestational diabetes, assuming that this will lower their risk of GDM. The design is presented in Figure 1. The Medical Ethics Committee of VU University Medical Centre at Amsterdam has approved the study design, protocols, and informed consent procedure. All participants must provide a written informed consent. After baseline measurements, participants will be randomly allocated to the control or intervention group. The participants will be followed for 9 months: from 15 weeks of pregnancy until 12 weeks after delivery.

### Study population

Participants will be pregnant women at increased risk for gestational diabetes. Women will be considered to be at increased risk for GDM if they are obese (BMI ≥ 30) or overweight (BMI ≥ 25) AND have at least one of the three following characteristics [21,22]:

1) history of macrosomia (offspring with a birth weight above the 97^th ^percentile of gestational age);

2) history of abnormal glucose tolerance during previous pregnancy;

3) first grade relative with diabetes mellitus type 2.

### Setting

This trial will be carried out in 10 midwifery practices and 3 larges hospitals in Amsterdam, The Netherlands. The midwives and obstetricians working in these practices or hospitals will invite women to participate in the study from October 2007 until December 2009.

### Inclusion and exclusion criteria

Pregnant women at increased risk for gestational diabetes will be eligible to enter the study when they meet the following inclusion criteria: between 14 and 20 weeks of pregnancy; over 18 years of age; sufficiently fluent in Dutch; being able to be moderately physically active; and willingness to give written informed consent.

Women will not be admitted to the RCT if they meet any of the following exclusion criteria: diagnosed with (gestational) diabetes mellitus before randomisation; hypertension (systolic pressure > 160 mmHg and/or diastolic pressure > 100 mmHg); alcohol abuse (i.e. 2 glasses alcohol or more per day); drug abuse (except for incidental analgesic agents); use of the medication that affects insulin secretion or insulin sensitivity (antiviral, corticosteroids, antihypertensive drugs, all concomitant medication will be discussed); serious pulmonary (COPD, exercise-induced asthma), cardiac, hepatic or renal (serum creatinine < 150 μmol/l) impairment; malignant disease; serious mental or physical impairment i.e. preventing to understand or implement the study protocol/aim.

### Recruitment

Women will be recruited at the Departments of Obstetrics of VU University Medical Centre (VUMC), St. Lucas Andreas Hospital (SLAZ) and Onze Lieve Vrouwe Gasthuis (OLVG) in Amsterdam and 10 midwifery practices in Amsterdam. The obstetricians or midwives in the 3 hospitals and midwifery practices will introduce the study at the first visit of every pregnant woman who is eligible for inclusion. They will give the pregnant women who are interested to participate a brochure about the study to read at home. Obstetricians and midwives will also fill out a reply coupon (name, telephone number, inclusion and exclusion criteria) and will send this coupon to the research team. Interested women can return the informed consent form to the research team or the research team calls within two weeks after receiving the reply coupon. When women meet the inclusion criteria and want to participate, they will be included. After inclusion, an appointment for the baseline measurement will be made.

### Sample size

Power analysis is based on the effects of the intervention program on fasting plasma glucose. A difference of 0.4 mmol/l in fasting plasma glucose between the two research groups is considered to be clinically relevant. Assuming a standard deviation of 0.7 mmol/l, this difference can be detected with a power (1-β) of 0.80 and an alpha of 0.05 with two groups of 64 subjects. Taking a loss to follow up of about 20% into account, a total of 160 subjects will be recruited. A significant difference of 300 grams in birth weight can also be detected with this number of participants, assuming a standard deviation of 600 grams.

### Randomisation

A computerized random number generator draws up an allocation schedule pre-stratified for the hospital where participants will be measured or where they will follow the exercise program; within hospitals respondents will be allocated at random to either the intervention or to the control group. Block randomisation in blocks of four will be performed. After baseline measurements, a research assistant will inform the women about the group they will be allocated to.

### Blinding

The key of coding concerning group assignment will only be known to the programmer of the database. The researcher and research assistant will not be blinded for allocation after randomisation. All outcome measures will be assessed by independent examiners, unaware of group allocation. Participants cannot be blinded for the intervention, but will be asked not to reveal information about their treatment to the examiners. Also obstetricians and midwifes cannot be blinded for the allocated treatment. Analyses will be performed blind for treatment allocation.

### Intervention

#### Intervention Group

Participants randomised to the intervention group will complete an exercise program on two days of the week during the remaining duration of the pregnancy. Each session will last for 60 minutes. The exercise program will consist of aerobic and strength exercises that will help to control the blood glucose level. All sessions will be completed under the guidance and supervision of a specifically trained physiotherapist. The sessions will be located at the Department Physiotherapy of the participating hospitals (VUMC, OLVG and SLAZ).

At the first session the participants will be introduced to the exercise program. They will be informed about the structure of the sessions and will receive instructions in the use of the equipment. Each exercise session will begin with a warming-up for 5–10 minutes. This is a light intensity activity, such as slow cycling at a level of intensity of 50 Watt, that prepares the muscles for exercise. After the warming-up, each participant completes an individualised program of 40 minutes, consisting of 1 or 2 aerobic exercises and 4 to 6 strength exercises. After exercising, participants cool down for 5–10 minutes by slowly reducing the activity. This allows the heart rate to return to normal levels.

**Figure 2:**
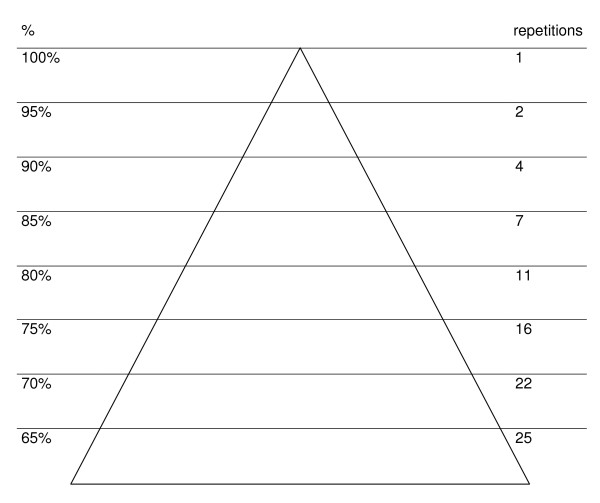
**Oddvar Holten diagram**.

**Figure 3:**
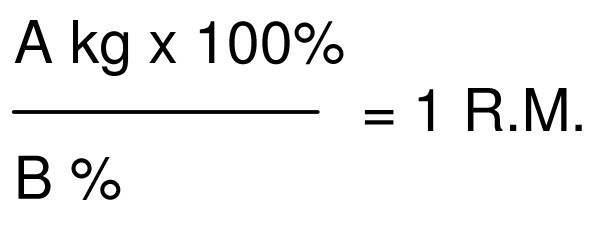
**Formula**. A: lifted weight; B: percentage of intensity.

At the start of the intervention exercises will be adjusted to maximal muscle strength and aerobic capacity of the women. However, during pregnancy it is not possible to determine maximal muscle strength and aerobic capacity levels with standard physical tests because of the pregnancy related physiological changes. The one Repetition Maximum (1-RM; the heaviest weight that can be lifted only once) weight lifting test to measure muscular strength cannot be used during pregnancy. Woman should avoid overly vigorous activity, because most pregnant women have a decreased tolerance for weight-bearing exercise and they should avoid the Valsalva maneuver during resistance exercise [7,23]. Therefore 1RM for each strength exercise will be predicted from the Oddvar Holten diagram [24]. In short this prediction is done as follows: the physiotherapist makes an estimation of the weight that can be lifted for 10–20 times; the number of repetitions that can be maximally performed is registered; the percentage of intensity can than be looked up in the Oddvar Holten diagram at the number of repetitions (figure 2) and 1RM can be computed by the formula (figure 3). For example, if a participant makes 16 repetitions with 24 kilos [i.e. 75% of 1 RM in the diagram] than, divide 24 by 0.75 for a 1RM of 32 kilos. To start an exercise program for example at 30% of 1RM: multiply the 1RM by 30%; in this case 32 times 0.30 equals 9.6 kilos. Consequently, participants will start exercising at a training load of 30% of 1RM with 15 repetitions, for 3 sets. Exercises will progress by first increasing the number of repetitions (from 15 to 30) and than by increasing the number of sets of repetitions (from 3 to 5). The training load will be increased (from 30 to 60% of 1RM) when the desired number of (sets of) repetitions with the current load is reached. For strength training, participants will use free weights and different machines to target major muscle groups in the upper body, thrunk and lower body.

ACSM recommends to not do a maximal aerobic exercise test in nonclinical settings during pregnancy [25]. During pregnancy maximal heart rate reserve decreases and resting heart rate increases. This makes the standard training HR formulas ineffective [25]. ACOG and Canadian guidelines therefore recommend the use of ratings of perceived exertion (RPE) in addition to heart rate [23,26]. Borg's conventional 6–20-point scale is recommended, with 12 to 14 (a rating of 13 corresponds to a subjective rating of "somewhat hard") identified as the RPE range to apply in pregnancy [27,28]. The PARmed-X form pregnancy document modified the target HR zones for exercise prescription from the suggested target HR zones for nonpregnant individuals. The target HR zone suggested for women aged 20–29 yr is 135–150 bpm and for women aged 30–39 yr 130–145 bpm [29]. These target HR zones during pregnancy represent approximately 60–80% of aerobic capacity based on age [23]. A check to avoid overexertion will be the "talk test". The exercise intensity is too high if the exercising pregnant woman is not able to carry on a verbal conversation [23,29]. Aerobic training will be executed on cycle ergometers, treadmills, cross-trainers, and when still possible with the pregnant belly, on stationary rowing machines. For every aerobic exercise a baseline level of intensity will be estimated. This prediction will be done as follows: for example a woman starts cycling at a low work rate of 50 watt with a pedal rate at 50 rpm. The physiotherapist increases the work load till an intensity is reached that equals a Borg scale rating of 12. Woman than maintain cycling for 10 minutes on that intensity. Exercises in the subsequent session will start on that baseline intensity. When the RPE is rated under 12, the intensity will be increased by increasing the working load. At each training, attendance and the intensity of all strength and aerobic exercises will be registered by the physiotherapist.

Training will take place under close supervision of a physiotherapist. The physiotherapists will have expertise with providing training for pregnant women. The physiotherapists will use the guide of the American College of Obstetricians and Gynaecologists [26,30] to maintain a safe and healthy exercise program. Warning signs to terminate exercise are; vaginal bleeding, dyspnoea before exertion, dizziness, headache, chest pain, muscle weakness, calf pain or swelling, preterm labour, decreased foetal movement, amniotic fluid leakage [26]. Furthermore, during training classes women will be encouraged to be physically active in their daily live also. Walking and cycling are activities that can be maintained up until delivery, and these are easily incorporated into daily live. To motivate the women for daily physical activity, information on the benefits for mother and child will be given at the start and during the intervention. If indicated, women in the intervention group will be treated as usual for GDM or other complications during pregnancy.

#### Control group

The control group will receive usual care given by obstetricians and midwives. Dutch midwives and obstetricians follow closely the health status of each pregnant woman and their unborn child. The first appointment is usually between 9^th ^and 12^th ^week of gestation. For women who develop GDM during the study, usual care consists of dietary advice and insulin treatment. Every treatment will be registered in the medical records.

#### Non-participants

Women who decide not to participate after reading the information brochure at home are asked to return a reply coupon. They are asked to fill in weeks of pregnancy, body weight, body height and reason for not participating. Characteristics of this group will be compared to those women who do participate in the study.

### Outcome measurements

Participants in both intervention and control group are invited for three measurement appointments during pregnancy and one appointment after pregnancy (see Table 1). Outcome measurements are assessed at baseline (around 15 weeks of pregnancy), 24 and 32 weeks of pregnancy and 12 weeks postpartum by means of physical measurements, laboratory tests, and self-administered questionnaires.

**Table 1:** Schedule of measurements.

**T0: 15 weeks pregnancy**	**T1: 24 weeks pregnancy**	**T2: 32 weeks pregnancy**	**T3: 12 weeks post partum**
Fasting blood sample	OGTT*	OGTT	Fasting blood sample
	Ultrasound*	Ultrasound	Neonatal weight**
Maternal weight	Maternal weight	Maternal weight	Maternal weight
Accelerometer	Accelerometer	Accelerometer	Accelerometer
Questionnaire	Questionnaire	Questionnaire	Questionnaire

*measurement as part of usual care in hospital; **weight of the baby at delivery, as part of usual care.

*Primary maternal outcome *measures are fasting plasma glucose and relative increase in insulin resistance. *Primary neonatal outcome *is birth weight.

*Secondary outcome *measures are: maternal serum triglycerides, HDL, cholesterol and HbA1c, maternal weight gain during pregnancy, maternal physical activity level, foetal growth. Changes in direct health care and non-health care costs and indirect non-health care costs are studies as well.

Although the groups are too small to be able to detect statistically significant changes in these outcomes, the following variables will be assessed as well because of their clinical importance: incidence of gestational diabetes, macrosomia (97^th ^percentile for gestational age), and complications during pregnancy or delivery, such as preeclampsia, caesarean section rate and need for labour induction. These outcomes will be collected from the medical records of the gynaecologist or midwife.

### Data collection

#### Physical measurements

##### • Maternal anthropometric measures

Maternal body weight gain will be measured by calibrated electronic scales (SECA 888) while participants are only wearing indoor clothing. Body weight will be measured twice, and the mean value of the two measurements will be computed.

Maternal body height will be measured on bare feet by a (wall mounted) height scale.

##### • Foetal and neonatal anthropometric measures

Foetal growth during the pregnancy will be measured by ultrasound. Obstetrical ultrasound will be used to assess the growth of the foetus. It will be performed by a sonographer of the hospital at 24 and 32 weeks of pregnancy.

Birth weight: At delivery, birth weight will routinely be measured and recorded by the obstetrician, midwife or the nurse. We will gather the birth weight from the medical records of the obstetrician of midwife.

##### • Physical activity

Physical activity will be measured by an accelerometer. At 15, 24 and 32 weeks of pregnancy and 12 weeks after delivery an accelerometer will be sent to all participants. They have to wear the accelerometer (Actitrainer) for 4 consecutive days to gather, objective data on the amount of daily physical activity.

#### Questionnaire

• Physical activity will be assessed with the Activity Questionnaire for Adolescents & Adults (AQUA) [31].

• Quality of life will be assessed with the EuroQol [32,33].

• All costs related to pregnancy and delivery: direct health care and non-health care costs and indirect non-health care costs. Examples of direct health care costs are costs for visits to the general practitioner, medical specialists, therapists, dieticians, costs of medication and hospitalisation. Examples of direct non-health care costs are the costs of over-the-counter medication, travel time, and waiting time. Indirect costs will include the costs of absence from paid and unpaid work. Costs will be calculated by multiplying the volume of resource use by cost prices, following the Dutch guidelines for economic evaluations in health care to estimate costs [34].

• Potential confounding variables such as age, ethnicity, socio-economic status, working status, and parity will be measured also.

#### Laboratory tests

##### • Fasting blood samples

Fasting blood will be drawn from the antecubital vein after a participant has fasted for at least 10 hours. From this blood samples glucose, HbA1c, HDL-cholesterol, triglycerides and cholesterol will be measured. HDL-cholesterol, triglycerides and cholesterol will be measured by means of enzymatic techniques (Boehringer-Mannheim, Mannheim, Germany). HbA1c will be measured by High Performance Liquid Chromatography. Plasma glucose will be measured in plasma by means of a hexokinase-method (Roche Diagnostics GmbH, Mannheim, Germany).

##### • Oral Glucose Tolerance Test (OGTT)

An OGTT will be performed after a fasting period of at least 10 hours. Blood samples will be collected in the fasting state and 30, 60, 90, 120 and 180 minutes after ingestion of a glucose drink (100 g D-glucose in 0.5 l water). Participants should not eat anything or do any physical activity or exercise during the test. The insulin sensitivity index will be calculated from a 100-g OGTT according to the equation derived by Matsuda and DeFronzo [35] in which insulin sensitivity is estimated by using the fasting and mean glucose and insulin concentrations in serum and HOMA-index. Simple fasting method to measure insulin resistance is the homeostasis model assessment (HOMA) (= fasting insulin concentration (μU/mL) × fasting glucose concentration (mmol/L)/22.5)) [36].

### Process evaluation

To assess factors that contribute to the success or failure of the program, a process evaluation will be performed. Trainers and a subgroup of 20 women will be interviewed about their experiences with the exercise program. Attendance to the exercise sessions will be recorded by the physiotherapists. Furthermore, 10 women who have declined to participate in the RCT will be interviewed about their reasons for declining, and their possible barriers towards participating in an exercise program.

### Statistical analysis

Longitudinal, linear regression analyses will be performed. In these analyses, the correlation between multiple measurements within one individual is taken into account. The regression coefficient reflects the average difference in the outcome variable between conditions. Possible confounders will be entered into the regression analyses. Confounding will be defined as a change in the regression coefficient of 10% or more. Possible effect modification will be studied as well, defined as a significant (p < 0.10) interaction-term between group allocation and the variable concerned. Data will be analysed according to the intention-to-treat principle.

### Cost-utility analysis/Cost-effectiveness analysis

The cost-effectiveness analysis will consist of two components. 1) A bootstrap technique will be used to estimate the costs from a patient and health insurer's perspective. 2) A detailed cost-utility analysis, using a decision analytic model will be performed to estimate costs associated with primary and secondary outcomes. Ad 1) Differences in mean costs between both groups will presented with 95% confidence intervals estimated using bootstrapping methods [37]. Cost-effectiveness and cost-utility ratio's will be assessed from a patient's and health insurer's perspective. All direct and indirect costs will be taken into account. Differences in direct, indirect and total costs between groups will be computed with 95% confidence intervals by using bootstrapping methods. Cost-effectiveness planes and acceptability curves will be presented [38]. Ad 2) For the cost-utility analysis, a linear decision tree will be modelled using the TreeAge Pro Healthcare Module. Pathophysiological, quality of life and cost data from this study will be supplemented by data from the literature. The model will be build based on the same methodology as described in the papers by Herbst [39] and Culligan [40], in which decision analyses on macrosomia are discussed.

## Discussion

The incidence of GDM is about 7% in the USA, but no accurate data on the incidence in the Netherlands are available. However, since the prevalence of overweight and obesity is increasing strongly as a consequence, an increase in the incidence of GDM is expected. Preventive strategies for women at risk for GDM are therefore of great importance. In the FitFor2-study, women with a high risk of developing GDM are identified, and given an exercise intervention, with the aim to prevent the occurrence of GDM, and perhaps also diabetes in later life.

The prevention of GDM is also relevant for the children, since it also puts the infant at risk. GDM is associated with increased risk for macrosomia, jaundice and birth trauma. Later in life, children of gestational diabetic mothers have an increased risk for obesity, abnormal glucose tolerance, and diabetes [6].

If the FitFor2 intervention program proves to be effective, pregnant women will benefit from the exercise program and a reduced risk for GDM. Obstetricians and midwives should consequently refer women at risk for GDM to a special exercise program. Implementation of the physical activity program in the Netherlands is relatively easy, since exercise programs for pregnant women under supervision of an experienced trainer are already available. These programs may have to be adjusted slightly to the FitFor2-program. Furthermore, the costs of these programs should be refunded by including them in the insurance policies.

## Abbreviations

GDM: gestational diabetes mellitus; CDC: Centers for Disease Control and Prevention; ACSM: American College of Sports Medicine; ACOG: American College of Obstetricians and Gynaecologists; RCT: randomised controlled trial; BMI: body mass index; VUMC: VU University medical centre; OLVG: Onze Lieve Vrouwe Gasthuis; SLAZ: Sint Lucas Andreas Hospital; RM: Repetition Maximum; RPE: Rate of Perceived Exertion; OGTT: oral glucose tolerance test; HOMA: homeostasis model assessment; AQUA: activity questionnaire for adolescents & adults.

## Competing interests

The authors declare that they have no competing interests.

## Authors' contributions

NO is responsible for data collection and wrote the manuscript. MvP originated the idea for the study, led on its design, and supervised the project. All authors participated in discussing the design of the FitFor2-study and developing the research protocols. All authors read and corrected draft versions of the manuscript and approved of the final manuscript.

## Pre-publication history

The pre-publication history for this paper can be accessed here:


